# Development and validation of warning system of ventricular tachyarrhythmia in patients with heart failure with heart rate variability data

**DOI:** 10.1371/journal.pone.0207215

**Published:** 2018-11-14

**Authors:** Wan-Tai M. Au-Yeung, Per G. Reinhall, Gust H. Bardy, Steven L. Brunton

**Affiliations:** 1 Department of Mechanical Engineering, University of Washington, Seattle, WA, United States of America; 2 Seattle Institute for Cardiac Research, Seattle, WA, United States of America; California State University Long Beach, UNITED STATES

## Abstract

Implantable-cardioverter defibrillators (ICD) detect and terminate life-threatening ventricular tachyarrhythmia with electric shocks after they occur. This puts patients at risk if they are driving or in a situation where they can fall. ICD’s shocks are also very painful and affect a patient’s quality of life. It would be ideal if ICDs can accurately predict the occurrence of ventricular tachyarrhythmia and then issue a warning or provide preventive therapy. Our study explores the use of ICD data to automatically predict ventricular arrhythmia using heart rate variability (HRV). A 5 minute and a 10 second warning system are both developed and compared. The participants for this study consist of 788 patients who were enrolled in the ICD arm of the Sudden Cardiac Death–Heart Failure Trial (SCD-HeFT). Two groups of patient rhythms, regular heart rhythms and pre-ventricular-tachyarrhythmic rhythms, are analyzed and different HRV features are extracted. Machine learning algorithms, including random forests (RF) and support vector machines (SVM), are trained on these features to classify the two groups of rhythms in a subset of the data comprising the training set. These algorithms are then used to classify rhythms in a separate test set. This performance is quantified by the area under the curve (AUC) of the ROC curve. Both RF and SVM methods achieve a mean AUC of 0.81 for 5-minute prediction and mean AUC of 0.87–0.88 for 10-second prediction; an AUC over 0.8 typically warrants further clinical investigation. Our work shows that moderate classification accuracy can be achieved to predict ventricular tachyarrhythmia with machine learning algorithms using HRV features from ICD data. These results provide a realistic view of the practical challenges facing implementation of machine learning algorithms to predict ventricular tachyarrhythmia using HRV data, motivating continued research on improved algorithms and additional features with higher predictive power.

## Introduction

Implantable Cardioverter–Defibrillator (ICD) therapy decreases mortality in select post-myocardial infarction and congestive heart failure (CHF) patients as shown in previous studies.[1, 2] However, ICD therapy does not solve many of the major problems associated with sudden cardiac arrest. ICDs do not prevent life-threatening cardiac arrhythmia (LTCA), but instead terminate arrhythmias after they occur. As a result, this can potentially cause harm to patients and their surroundings if, for example, they are driving, biking or in a situation where they can fall. It would be ideal for ICDs to issue a warning for impending ventricular tachyarrhythmia so that a patient can refrain from such activities or situations and healthcare providers can be alerted. Also, ICD shocks affect a patient’s quality of life and psychological state as they can cause anxiety, fear and excessive worry.[3,4] In particular, ICD shocks are very painful. The ICD shock sensation has been described as a strong kick in the chest and is rated a “6” on a 0 to 10 pain scale.[5, 6] Moreover, ICD shocks may be applied inappropriately in situations where the arrhythmia is not present. Therefore, if the ICD can predict the occurrence of ventricular tachyarrhythmia, this may provide a means to avert the shock and an opportunity for developing alternative approaches, such as preventive pacing.

From past research, there is evidence that a patient’s autonomic tone plays a role in the arrhythmogenesis of ventricular arrhythmia.[7, 8] Thus, it may be possible to predict the occurrence of ventricular arrhythmia by analyzing the heart rate variability (HRV) signals. It may also be possible to avoid the occurrence of ventricular arrhythmia by modulating the autonomic tone, though that remains to be investigated.[9]

Machine learning has been increasingly used in medicine in recent years.[10, 11] For example, it was advocated for prediction of patients’ risks for different diseases.[12] In particular, HRV signals have been used with machine learning to predict the occurrences of sudden cardiac death (SCD) in the past.[13–18] However, most of these studies compared pre-ventricular-tachyarrhythmia signals from human subjects at risk of SCD with HRV signals from healthy human subjects. While those findings show that there are significant differences between pre-ventricular-arrhythmia HRV signals from patients with heart disease and HRV signals from healthy subjects, we cannot conclude how practical it is to predict ventricular tachyarrhythmia in patients who received ICDs. In order to predict ventricular tachyarrhythmia in patients, the ICDs need to distinguish pre-ventricular-tachyarrhythmia rhythms from regular rhythms from the same patients. In the landmark Sudden Cardiac Death–Heart Failure Trial (SCD-HeFT), there were pre-ventricular-tachyarrhythmia rhythms and regular rhythms collected from a group of patients with congestive heart failure (CHF). In this paper, we used this data to develop and validate our prediction model of the onset of ventricular tachyarrhythmia in patients with congestive heart failure with machine learning algorithms.

## Methods

### Subjects

The Sudden Cardiac Death–Heart Failure Trial (SCD-HeFT) was a large National Institutes of Health funded multicenter clinical trial that was designed to study the effectiveness of ICD and amiodarone therapies in patients with mild to moderate heart failure.[1] Patients were randomly assigned in equal proportions to receive placebo (n = 847), amiodarone (n = 845) or a single-chamber ICD programmed to shock-only mode (model 7223, Medtronic) (n = 829) from September 16, 1997 to July 18, 2001. All patients were followed until October 31, 2003. The inclusion criteria for the study was the following: the patients had to be at least 18 years old, have NYHA class II or III chronic, stable CHF due to ischemic or nonischemic causes and a left ventricular ejection fraction (LVEF) of no more than 35 percent. The protocol was approved by the University of Washington institutional review board, and each patient was provided written informed consent before enrollment. The study sample for this paper (n = 788) is a proportion of the patients enrolled in the ICD arm (95.1% of n = 829) in SCD-HeFT and their demographics are listed in Table 1. Some patients were excluded because their records of HRV data did not satisfy the inclusion criteria outlined below in the section of HRV data.

**Table 1:** Demographics of patients in the sample (n = 788).

Age at Enrollment	Median: 60 years old Interquartile Range: 51–69 years old
Sex	609 Males 179 Females
Race	615 Caucasians 137 African Americans 26 Latin Americans 9 Asians 1 Native American
NYHA Functional Class	Class II: 541 Class III: 247
Cause of Heart Failure	Ischemic: 412 Non-ischemic: 376
6-minute Walking Distance (ft)	Median: 1133 Interquartile Range: 840–1350
Beta-blocker	Yes: 548 No: 240
LVEF (%)	Median: 24 Interquartile Range: 19–30
Handedness	Left: 77 Right: 711

### ICDs

SCD-HeFT included a pre-specified protocol for ICD programming.[1] This included a single zone of therapy at a detection rate of ≥187 beats/min. The initial detection interval was for 18 of 24 beats, and the redetect interval was for 12 of 16 beats. Shock-only mode was used hence there was no antitachycardia pacing. Antibradycardia pacing was set to 50 beats/min with hysteresis of 34 beats /min, which is the minimal allowed heart rate. The ICD has a sampling rate of 128Hz for reading the ECG of the patient. It also can store up to 2048 R-R intervals.

### HRV data

Patients with ICDs enrolled in SCD-HeFT went to the clinic to have 25–30 minutes of their HRV data downloaded on-site every three months throughout the trial. ICDs also stored the last 2048 R-R intervals and intracardiac electrograms that immediately preceded the occurrences of shocks. Later, these ECGs were examined by a blind events committee in SCD-HeFT to determine whether the shocks were appropriate or inappropriate. Based upon their decision, the HRV data that preceded the shocks were then classified into the appropriate shock group or the inappropriate shock group. Of the total 11764 records, only those that have 1700 or more normal beats, belonging to regular rhythms and pre-appropriate-shock rhythms, are included in our analysis, totaling 6890 records. This minimum number of 1700 normal beats is set as an inclusion criteria to provide enough HRV data for processing. The HRV data were extracted by the ICDs. These HRV data were examined to exclude premature ventricular contractions and compensatory pauses. Only normal beats were included during the final analysis.

Based on the inclusion criteria of the HRV data, 41 out of 829 patients who were enrolled in the ICD arm of SCD-HeFT had to be excluded from this study. Among patients included in this study, on average 3 ventricular tachyarrhythmia occurred in 10 patients and on average there were 8.5 records of regular rhythms downloaded from each patient over the time period examined.

### HRV processing and feature extraction

The goal of this paper is to predict the imminent onset of ventricular tachyarrhythmia. Therefore, we analyzed the HRV data up to 5 minutes before shocks and up to 10 seconds before shocks to identify a signature of the onset of VF/VT and to compare the accuracy of these prediction. If accurate prediction can be made 10 second before the occurrences of ventricular tachyarrhythmia, perhaps an innovative and preventive pacing can be triggered in the ICDs to avoid the occurrences of ventricular tachyarrhythmia.

The methods used for feature extraction from the HRV data are described below.

#### Principal component analysis (PCA)

PCA is cornerstone of dimensionality reduction and statistical data analysis.[19,20] For multi-dimensional data, such as the features extracted from HRV data, or for time-series of HRV data, PCA identifies a new hierarchy of features given by orthogonal combinations of features that are most correlated in the data. The new features are known as principal components (PCs), which are ordered based on the variance they capture in the data. PCA is especially useful to analyze high dimensional data. With PCA, one can retain the dominant features while discarding the least informative components. Thus, PCA simultaneously reduces the dimension of the feature space while also denoising the data. PCA has been applied broadly to electrocardiogram (ECG) signals [21–24] and to HRV data to assess cardiac autonomic neuropathy.[25] A combination of PCA and least squares SVM has also been used to detect ECG arrhythmia.[26]

In this work, the time-series of HRV data for each patient sample are shaped into column vectors which are then arranged into an *m*×*n* matrix R, where *m* is the number of R-R intervals in each of the recordings (1000 for 5-minute prediction and 1600 for 10-second prediction) and *n* is the number of recordings. The mean of all columns is subtracted from each column, and the columns are then divided by their standard deviation so that each column has unit variance. Then the reduced singular value decomposition (SVD) of the matrix is computed:

$$R=U\times S\times V^{T}$$ (1)

In (1), U and V are unitary matrices while S is a diagonal matrix containing the singular values in decreasing order along its diagonal. U×S encodes the PCs and V determines the magnitude of the projection of the data onto the PCs.

Choosing the right number of singular values to keep is one of the most crucial decisions when using the SVD. Here, we used the method of Gavish and Donoho to determine the optimal number of principal components for the purpose of training the classifiers and prediction.[27]

#### Mean N-N interval

The mean N-N interval of each recording is calculated and used as a candidate predictor for ventricular arrhythmias.

#### Hjorth complexity and Hjorth mobility

In 1970, Bo Hjorth introduced the Hjorth parameters (Hjorth activity, Hjorth complexity and Hjorth mobility) which are indicators of statistical properties of signals in the time domain.[28] Hjorth activity is the signal power, Hjorth mobility gives an estimate of the mean frequency, and Hjorth complexity represents an estimate of the bandwidth of the signal. In particular, Hjorth complexity and Hjorth mobility were used previously to predict SCD with promising results.[29] Therefore, in this study, Hjorth mobility and Hjorth complexity of the HRV signals are used as features to predict ventricular tachyarrhythmia.

#### Detrended fluctuation analysis (DFA)

DFA is a predictive tool for heart disease introduced by Peng et al.[30] The DFA yields the short-term and long-term fractal scaling exponents, α_1_ and α_2_ respectively, which are measures of the self-similarity of the HRV data in time. DFA is suited for analyzing non-stationary physiological signals because it discards changes that are due to external environmental stimuli and focuses on the intrinsic dynamics of the heart itself. The fractal scaling exponents α_1_ and α_2_ have been considered prognostic in certain disease states.[31,32] In performing DFA, all HRV data were analyzed using the “fastdfa” algorithm as given by Little et al.[33]

#### HRV signal in frequency domain

The HRV signals were analyzed in the frequency domain using the Lomb periodogram algorithm.[34–36] The Lomb periodogram method was chosen because HRV signals are sampled at uneven time intervals and this algorithm performs well in the presence of artifacts, pacing and premature ventricular contractions. The frequency range investigated is 0Hz to 0.5Hz. This frequency range was divided into five logarithmically equal spaced bins and the power of the HRV signals in each frequency domain bin was calculated using the Lomb periodogram.

### Machine learning

#### Process of machine learning

The features listed above (PCs, fractal scaling exponents, power in bins, etc.) were extracted from each record and were organized into a matrix. Each column of the matrix input corresponds to the same feature for all records. Each row contains the features for one record and is labeled as belonging to either the regular rhythm group or the pre-appropriate-shock rhythm group; the labeled data are used to train classifiers. There are many more records in the regular rhythm group (6660 records) compared with the appropriate shock group (230 records). In order to avoid bias created by an imbalanced dataset, we randomly selected samples from the regular rhythm group to match the number of samples in the pre-appropriate-shock rhythm group.[37] Then, we randomly selected 80% of the selected sample to be the training set for the machine learning algorithms. Once a classifier is constructed using the training dataset, it is tested with the remaining 20% of the selected sample (the test set) to investigate the classification performance. To estimate the out of sample error of the classification algorithm, the procedure was repeated 100 times. The HRV data were pre-processed to extract all the features except the PCs. PCA was performed and PCs were extracted independently in each training set.

The performance of the classification is measured by sensitivity, specificity and area under curve (AUC). Sensitivity is the percentage of recordings in the appropriate shock group (positive group) that the machine learning algorithm classifies correctly. Specificity is the percentage of recordings in the regular rhythm group (negative group) that the machine learning algorithm classifies correctly. AUC is the area under the receiver operating characteristic (ROC) curve which plots the sensitivity versus 1 –the specificity as the decision threshold varies.[38, 39]

#### Algorithms from machine learning

In this paper, two leading algorithms from machine learning, random forest (RF) and support vector machine (SVM), were trained and used to classify records in the test set in order to evaluate prediction performances using the features listed above.

A random forest (RF) is an ensemble learning method for classification.[40] It consists of a group of decision trees formed by the technique of bootstrap aggregating.[41] In addition, RFs select a random subset of the features at each candidate split in the learning process. To decide on the class of a new sample, each of the decision trees will output a class and the mode of these outputs determines the final decision of the RF. In training the RF classifiers, the importance of each predictor is calculated using the increase in prediction error if the values of that predictor are permuted across the out-of-bag records.

A SVM is a supervised learning model and its goal is to find a hyperplane that gives the maximum separation margin between the samples from the two classes of data, enabling accurate classification. Samples on the margin are called the support vectors.[42–44] SVMs with linear kernels were used in this paper using normalized data. The data is normalized by first subtracting the mean and then dividing the difference by the standard deviation.

## Results

Table 2 and Table 3 show the statistics of all the features except the PCs for regular rhythms and pre-appropriate-shock rhythms for 5-minute and 10-second predictions. The p-values were obtained by performing Mann-Whitney U-test.

**Table 2:** Statistics of features for 5-minute prediction.

	Regular Rhythms Median (Interquartile Range) (n = 6660)	Pre-appropriate-shock Rhythms Median (Interquartile Range) (n = 230)	p-values
*α*_1_	0.662 (0.459 to 0.999)	0.518 (0.349 to 0.638)	<0.001
*α*_2_	1.03 (0.798 to 1.18)	0.644 (0.502 to 0.930)	<0.001
Mean N-N interval (s)	0.802 (0.716 to 0.902)	0.694 (0.583 to 0.804)	<0.001
Power in bin 1 (s^2)	9.72e-5 (2.05e-5 to 3.55e-4)	3.14e-5 (9.44e-6 to 1.98–4)	<0.001
Power in bin 2 (s^2)	1.52e-3 (5.37e-4 to 3.37e-3)	4.38e-4 (1.62e-4 to 1.60e-3)	<0.001
Power in bin 3 (s^2)	1.43e-3 (7.26e-4 to 2.64e-3)	7.38e-4 (4.01e-4 to 1.56e-3)	<0.001
Power in bin 4 (s^2)	7.94e-4 (4.12e-4 to 1.62e-3)	1.17e-3 (4.64e-4 to 2.58e-3)	0.0217
Power in bin 5 (s^2)	6.97e-4 (2.62e-4 to 2.38e-3)	2.07e-3 (9.01e-4 to 7.54e-3)	<0.001
Hjorth complexity	1.71 (1.61 to 1.78)	1.75 (1.72 to 1.79)	<0.001
Hjorth mobility	0.0760 (0.0461 to 0.140)	0.168 (0.0974 to 0.252)	<0.001

**Table 3:** Statistics of features for 10-second prediction.

	Regular Rhythms Median (Interquartile Range) (n = 6660)	Pre-appropriate-shock Rhythms Median (Interquartile Range) (n = 230)	p-values
*α*_1_	0.670 (0.467 to 1.00)	0.519 (0.357 to 0.639)	<0.001
*α*_2_	1.04 (0.807 to 1.18)	0.645 (0.504 to 0.944)	<0.001
Mean N-N interval (s)	0.806 (0.720 to 0.906)	0.682 (0.576 to 0.799)	<0.001
Power in bin 1 (s^2)	6.00e-5 (1.20e-5 to 2.18e-4)	2.79e-5 (1.30e-6 to 1.57e-4)	<0.001
Power in bin 2 (s^2)	9.23e-4 (3.44e-4 to 2.26e-3)	4.47e-4 (1.23e-4 to 1.62e-3)	<0.001
Power in bin 3 (s^2)	1.11e-3 (5.35e-4 to 2.16e-3)	6.25e-4 (2.2e-4 to 1.39e-3)	<0.001
Power in bin 4 (s^2)	6.41e-4 (3.18e-4 to 1.33e-3)	7.72e-4 (3.52e-4 to 2.03e-3)	0.007
Power in bin 5 (s^2)	5.08e-4 (1.90e-4 to 1.88e-3)	2.49e-3 (7.60e-4 to 7.11e-3)	<0.001
Hjorth complexity	1.71 (1.60 to 1.78)	1.74 (1.70 to 1.78)	<0.001
Hjorth mobility	0.0752 (0.0436 to 0.142)	0.197 (0.118 to 0.274)	<0.001

**Fig 1:**
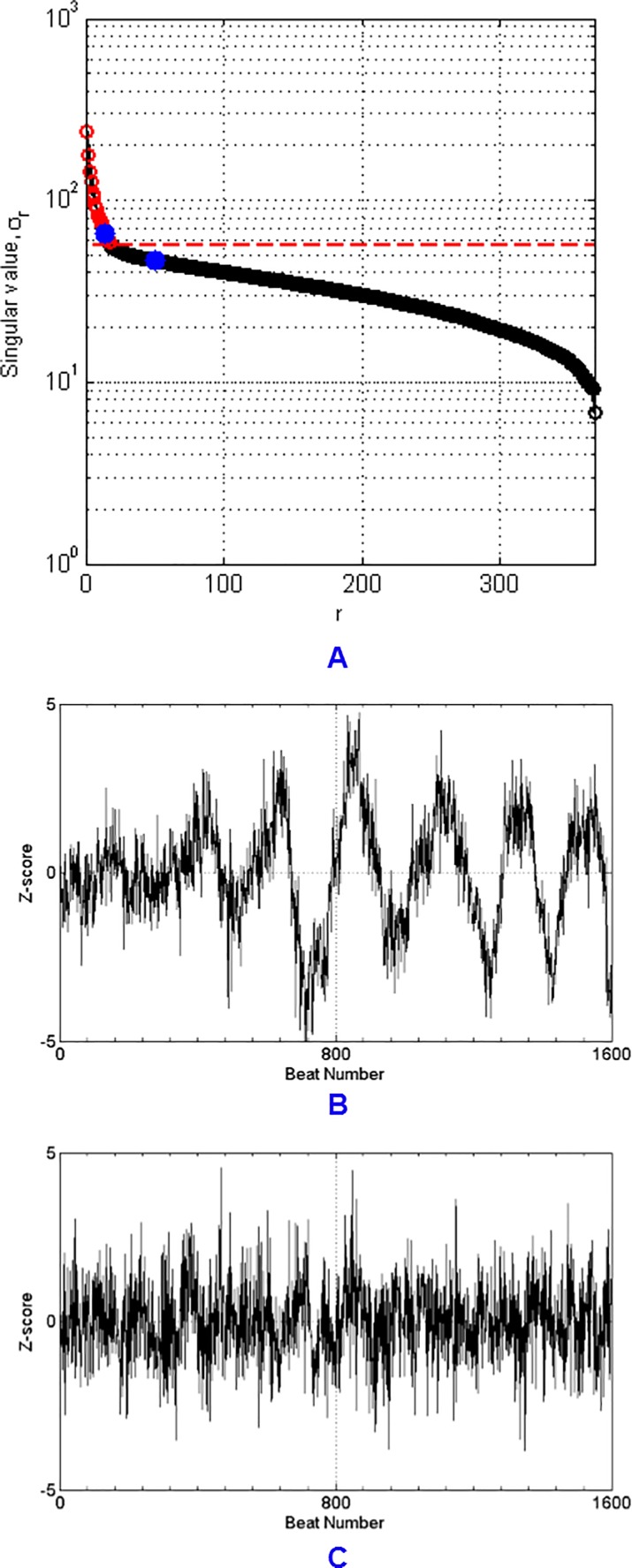
Plots of singular values and the principal components (PCs). (A) Plot of singular values with the optimal hard threshold found by the method proposed by Gavish and Donoho.^27^ The singular values that are above the threshold are plotted in red while the ones that are below the threshold are plotted in black. The blue dot above the red dash line corresponds to the 14^th^ singular value. The blue dot below the red dash line corresponds to the 50^th^ singular value. (B) PC 14. (C) PC 50.

Fig 1A shows a plot of singular values from PCA in log scale and shows the threshold above which the singular values contain worthwhile information, obtained from one of the training sets. It can be seen that PC 1 to PC 18 should be kept according to the Gavish Donoho criterion. The plots of PC 14 and PC 50 in Fig 1B and Fig 1C emphasize that above the threshold, PCs have discernable structure, while below the threshold, PCs are characterized by noise.

**Fig 2:**
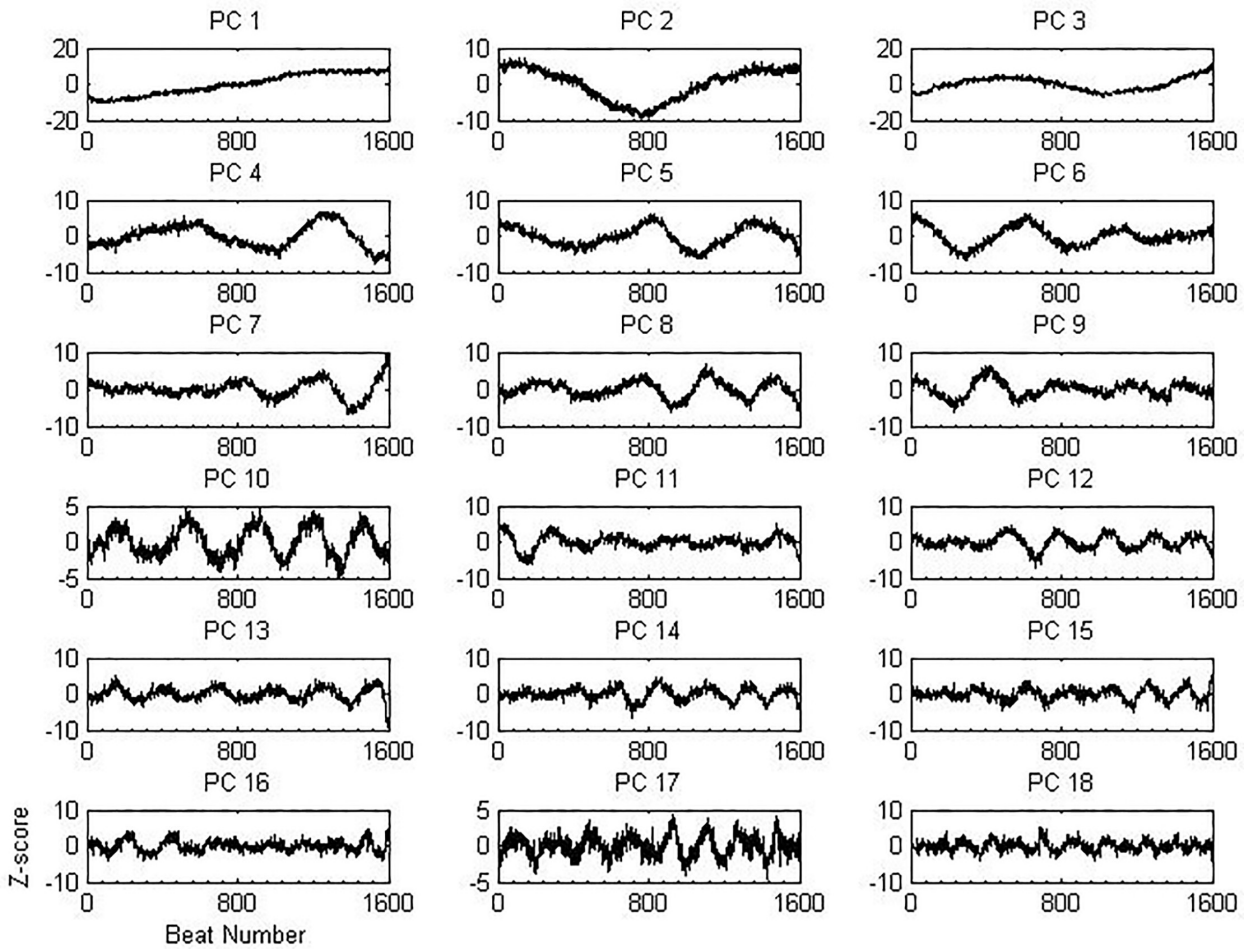
First 18 principal components of HRV data matrix from one of the training sets.

Fig 2 shows the first 18 PCs of regular rhythms and 10-second-pre-appropriate-shock rhythms from one of the training sets. These PCs resemble signals of different frequencies. In particular, PC 1 can be used to represent a steady increase in N-N interval over time. The first 18 PCs were included as features for the classifiers.

For PC 1, a positive coefficient indicates that the N-N interval gets longer over time. While a negative coefficient indicates otherwise. For the 10-second prediction, for one of the training sets (n = 184 for regular rhythms and n = 184 for pre-appropriate-shock rhythms), there was a significant decrease in the PC 1 coefficient for pre-appropriate shock rhythms as compared to regular rhythms (-0.008 [-0.042,0.009] vs 0.021 [-0.013, 0.054], p<0.001)

Table 4 shows the performances of 5-minute and 10-second prediction of ventricular tachyarrhythmia using RF and SVM. The accuracy of 10-second prediction is higher than that of 5-minute prediction. The AUC for 10-second prediction with RF and SVM is 0.87–0.88 while the AUC for 5-minute prediction with RF and SVM is about 0.81. In addition, the performance achieved with RF and SVM are comparable.

**Table 4:** Classification performance on test set for 5-minute and 10-second predictions using RF and SVM. 5-minute-pre-shock data contain 1000 R-R intervals while 10-second-pre-shock data contain 1600 R-R intervals. Each test was averaged over one hundred random cross-validation trials.

	5-minute prediction		10-second prediction	
	RF	SVM	RF	SVM
Sensitivity	0.74 (0.073)	0.75 (0.10)	0.79 (0.059)	0.80 (0.068)
Specificity	0.74 (0.065)	0.75 (0.12)	0.80 (0.064)	0.77 (0.063)
AUC	0.81 (0.046)	0.81 (0.077)	0.88 (0.035)	0.87 (0.037)
	Results are listed as mean (SD)			

**Fig 3:**
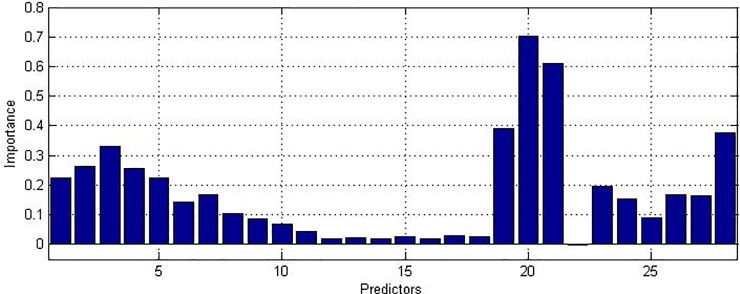
Average importance of predictors for 5-minute prediction. From predictor 1 to predictor 28: PC 1 –PC 18, α_1_, α_2_, mean N-N interval, power in 5 bins in frequency domain from low frequency to high frequency, Hjorth complexity and Hjorth mobility.

**Fig 4:**
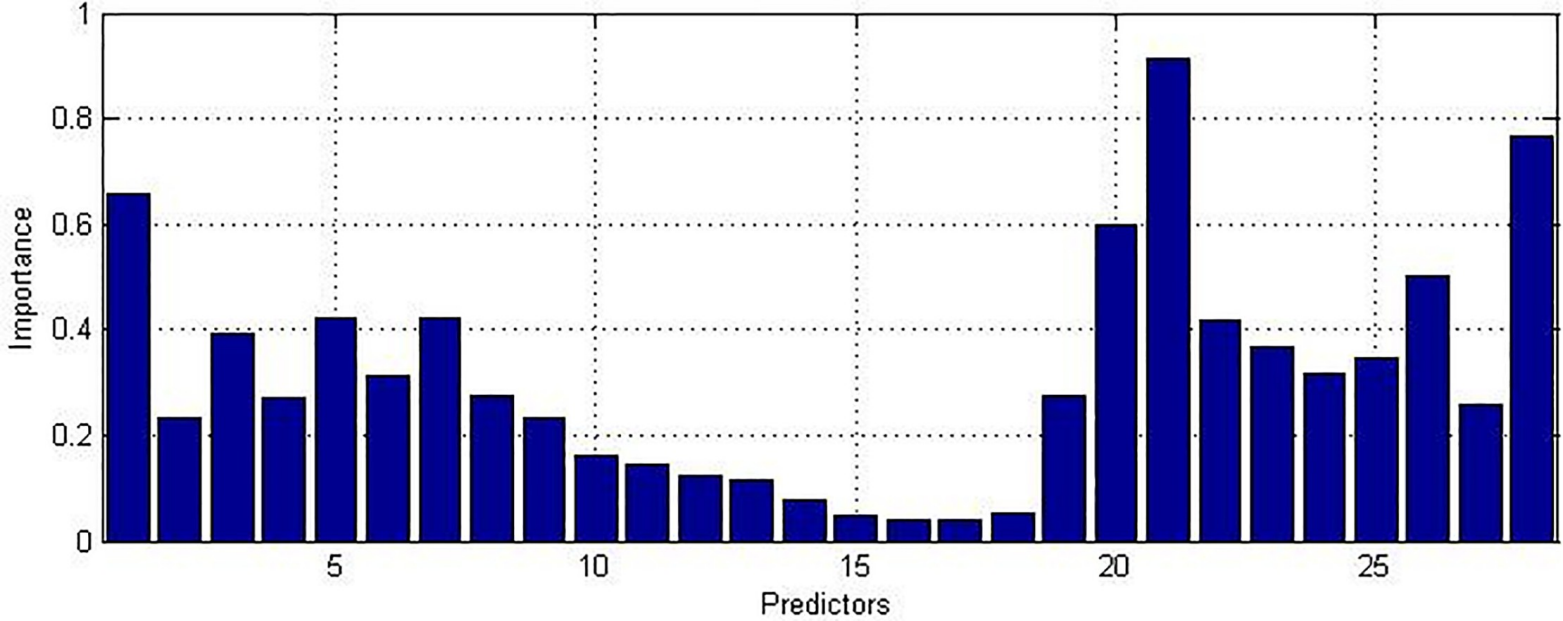
Average importance of predictors for 10-second prediction. From predictor 1 to predictor 28: PC 1 –PC 18, α_1_, α_2_, mean N-N interval, power in 5 bins in frequency domain from low frequency to high frequency, Hjorth complexity and Hjorth mobility.

From Fig 3, it can be seen that the most important predictor in 5-minute prediction of ventricular tachyarrhythmia is the long-term fractal scaling exponent, α_2_, followed by the mean N-N interval and then the short-term fractal scaling exponent, α_1_. Therefore, one can observe differences in the HRV signals in the nonlinear domain minutes before the occurrence of ventricular tachyarrhythmia. Fig 4 shows for 10-second prediction, the mean N-N interval is the most important predictor, followed by Hjorth mobility and PC 1. Therefore, one can conclude that as the occurrence of ventricular tachyarrhythmia approaches, the features of HRV signals in the time domain have the most predictive power. Even though features in the nonlinear domain are most important for 5-minute prediction and features in the time domain are most important for 10-second prediction, features in the frequency domain still hold important predictive power, as shown by their importance in Figs 3 and 4. Therefore, it is still worth including predictors in different domains for prediction to improve accuracy. It is also observed that the importance of PC 1 to PC 18 generally decreases. This is consistent with the associated singular values.

## Discussion

In this paper, we develop and validate a 5-minute and a 10-second warning system for ventricular tachyarrhythmia. We show that one can predict ventricular tachyarrhythmia with moderate accuracy using modern machine learning algorithms and a combination of features from HRV signals in time, frequency, and nonlinear domains in the same group of patients with heart failure. While there has been research on the prediction of SCD using HRV signals,[13–18] most of these previous studies use HRV signals from healthy subjects as the baseline to predict SCDs in patients with heart disease. Also, while there is a previous study on the prediction of ventricular tachycardia using pre-ventricular-arrhythmic rhythms and control rhythms from the same group of patients, the sample has 41 subjects and 104 recordings.[45] The results in the present paper give a realistic view of how practical it is to automatically predict ventricular tachyarrhythmias in ICD patients with heart failure with HRV data 5 minutes or 10 seconds before their occurrences.

Our results show that the accuracy is higher for a 10-second warning system than a 5-minutewarning system, which may suggest that it is easier to make a correct prediction when the ventricular arrhythmia draws nearer. Previous study has shown that higher heart rates are a predictor of cardiac arrest and death.[46] But we know of no previous study definitively proving a rise in heart rate in the minutes immediately prior to VT/VF in the general study population.

Using machine learning for real-time classification is extremely challenging on a number of fronts. First, practically speaking, these algorithms are generally computationally intensive: real-time processing would require significant computational resources and would also rapidly drain the battery of a device. Even if these challenges can be overcome, the algorithms themselves will need to improve to have the sensitivity and specificity required for continuous real-time monitoring. A life-threatening ventricular arrhythmia is a rare event, making it difficult to accurately predict without many false positives. However, the wealth of data collected during real-time continuous monitoring may dramatically improve our ability to accurately classify events in the future especially if adjustments could be made continuously to machine learning classifiers to improve sensitivity and specificity in a particular individual whose rhythm is being monitored continuously for months or years.

To detect early warning signs of life-threatening ventricular arrhythmias in real time, the most recent half an hour of HRV data should be classified continuously using a classifier that has already been trained on pre-appropriate-shock and regular rhythms from a large sample of subjects with heart failure. The prediction score from the classifier indicates the likelihood of an impending ventricular arrhythmic event. In this paper, RF and SVM classifiers were trained with an equal number of samples in the positive class (pre-appropriate shock rhythms) and negative class (regular rhythms) to avoid bias caused by skewed data. Classification performance was obtained by classifying test sets that had equal numbers of recordings in the positive and negative groups. However, if this algorithm is installed in patients’ ICDs, it will observe many more regular rhythms than arrhythmia. It certainly will misclassify some of the regular rhythms as arrhythmia and falsely trigger the warning system given that the specificity is about 75% for 5-minute prediction and 80% for 10-second prediction. Too many false warnings will disrupt the patients and medical units. This is a problem that must be addressed in the real-world implementation of the warning algorithm. One possible solution is to give warning only when the classification is made with high statistical confidence. In other words, it is possible to increase the decision threshold, above which a warning will be issued, in order to increase the specificity. However, the sensitivity will drop at the same time, which means that the classifier will miss more impending ventricular tachyarrhythmia. Another possible solution is to wait until clear precursors to ventricular tachyarrhythmia develop, such as heart rate increasing steadily for the past half an hour, before giving warnings of ventricular tachyarrhythmia. However, an earlier warning will enable patients to take precautions sooner and increase the readiness to respond. Nonetheless, the challenge of excessive false warnings will need to be addressed before the practical implementation of such predictive algorithms can be uniformly employed. The ultimate solution for improving the classification accuracy would be to add more features/predictors or to use more sophisticated machine learning algorithms while keeping the computational cost to a minimum.

In this paper, we only reported the results of classification of pre-appropriate-shock rhythms and regular rhythms. One other useful application of the proposed method would be the classification of pre-appropriate-shock rhythms and pre-inappropriate-shock rhythms. Though it is not reported in the main body of this paper, we examined this application and found that the classification results are not as accurate as for classifying between pre-appropriate-shock rhythms and regular rhythms. This suggests that the features examined were similar during pre-inappropriate-shock rhythms and pre-appropriate-shock rhythms. Future work should extract additional features that can capture the differences between pre-appropriate-shock and pre-inappropriate-shock rhythms.

One caveat of our dataset is that it was drawn from subjects who were enrolled in the SCD-HeFT, which has a very specific inclusion criteria of class II and III congestive heart failure. Ventricular tachyarrhythmias do not only occur in patients with heart failure, but in people who are at higher risk including a much broader demographic. It is unclear how these results from subjects with heart failure can be generalized to a broader population. However, we believe that the classifier can be generalized in concept to patients regardless of whether or not they have heart failure. Further to this point, we believe the use of anti-arrhythmic medication will not significantly alter the usefulness of this approach. This is because out of the 788 subjects in the sample, 548 subjects were taking beta-blocker medication, which may reduce HRV and yet, the findings were consistent across these subgroups.

In this study, we extracted and used features from HRV data. HRV is impacted by the central nervous system and represents a constellation of influences, such as exercise, anxiety or thyroid function. That said, our numbers are significant for a clinical trial of ICD therapy and mirror realistic circumstances in which cardiac arrest occurs, where one or more of the above-mentioned influences either trigger or are associated with the onset of a lethal arrhythmia. We believe that the features extracted from the HRV data are generalizable indicators, but we are not yet able to pinpoint the specific influential trigger, which is a limitation of this study.

Information extracted from HRV will lose validity in the case of abnormal atrioventricular (AV) node function due to AV nodal blocks or atrial tachyarrhythmia, such as atrial fibrillation (AF), where there is a significant variation in the R-R interval. By definition, patients with AV block were excluded from enrollment and therefore we cannot comment on this population. The incidence of AF was 17% at the time of enrollment in the ICD population. Our findings therefore incorporate a moderate AF sub-population but insufficient in number to confirm the value of this diagnostic in AF alone. Likely, HRV changes during AF would be more significant in shorter R-R interval variability, as the likely precipitant was simply rate acceleration from adrenergic or vagolytic influences. Nevertheless, the relatively small AF population size makes it difficult to draw meaningful conclusions in this subgroup.

Another limitation of this study is that only HRV data were available. A past study shows that parameters from respiratory rate variability (RRV) analysis may have higher predictive power.[45] In the future, parameters from HRV signals should be used in combination with parameters from other signals to provide prediction of ventricular tachyarrhythmia with higher accuracy.

Since the classifiers in this study were developed using a population of patients with a very specific demographic and ventricular arrhythmias occur in a much broader population, studies should be conducted in the future to include patients with a broader demographic so that a more generalizable VT/VF warning algorithm can be developed.

In this work, the machine learning algorithms classify rhythms based on patterns learned in the existing training dataset. It would be ideal if the classifier adapts and learns from individual patients’ records of HRV data to optimize performance. Exploiting the variability among different patients’ HRV data will be the subject of future work. In addition, since the ICD can also record the patient’s electrogram, leveraging this data in the future to extract predictive features may also improve accuracy of classification. SVM and RF classifiers were used because they are robust, perform well in most situations, and are easily implemented. One can certainly experiment with other algorithms from machine learning, such as one-class SVM which is effective for anomaly detection. Our results suggest that the classification accuracy increases when the ventricular arrhythmias draw nearer. To verify this claim, one should further develop different warning systems, such as a 2-minute warning system, to see if its performance falls between the performance of the 10-second and 5-minute warning systems. This is an important avenue of future work.

## Conclusion

We have shown that it is possible to predict the onset of VF/VT as much as 5 minutes prior to their occurrence in patients with moderate heart failure with moderate accuracy. Much improvement is needed to overcome the problem of excessive false warnings in the real-world application of such a system in a broader demographic. However, if such a machine learning algorithm is implemented successfully, the early warning capability will provide a major advance in ICD technology and a significant improvement in the treatment of patients as it improves patient safety and readiness, alerts healthcare providers, and, possibly provides a platform for ICDs to administer direct, preventative interventions or, at minimum, deploy emergency medical services. We trust this work will motivate more research in this area.
